# A Linarin Derivative Protects against Ischemia-Induced Neuronal Injury in Mice by Promoting Cerebral Blood Flow Recovery via KDELR-Dependent CSPG4 Activation

**DOI:** 10.1155/2022/6434086

**Published:** 2022-07-26

**Authors:** Cong Xie, Shiqin Yue, Xiangzhu Li, Zongyang Li, Weiping Li, Guodong Huang, Guoxu Ma, Wenlan Liu, Yachao Wang, Yuan Zhang

**Affiliations:** ^1^Department of Neurosurgery, Shenzhen University First Affiliated Hospital, Shenzhen Second People's Hospital, Graduate School of Guangzhou Medical University, Shenzhen University, Shenzhen 518035, China; ^2^School of Pharmaceutical Sciences, Health Science Center, Shenzhen University, Shenzhen 518035, China; ^3^Institute of Medicinal Plant Development, Chinese Academy of Medical Sciences and Peking Union Medical College, Beijing 100193, China

## Abstract

The cerebral ischemic microvascular response and collateral circulation compensatory capacity are important for the outcome of ischemic stroke. Here, we sought to evaluate the effect of a linarin derivate 4′-benzylapigenin-7-*β*-rutinoside (BLR) on neurological function and cerebral blood flow restoration in ischemic stroke. A mouse model of middle cerebral artery occlusion (30 min) with reperfusion (24 h) was used to mimic ischemic stroke *in vivo*, and 2,3,5-triphenyltetrazolium chloride (TTC) staining, terminal deoxynucleotidyl transferase dUTP nick end labeling (TUNEL) assays, and immunofluorescence microscopy were used to assess the protective effects of BLR on infarct volume, neurological function, neuronal apoptosis, and inflammatory damage. Cerebral blood flow was assayed by laser speckle contrast imaging. Double immunostaining of GFAP-collagen IV and brain lucidification were performed to determine the protective effects of BLR on the disruption of brain vasculature. Differential gene expression was assessed by RNA sequencing. Coimmunoprecipitation and western blotting were used to explore the mechanism of BLR-induced neuroprotection. The results of *in vivo* experiments showed that BLR administration after reperfusion onset reduced infarct volume, improved neurological function, and decreased the neural cell apoptosis and inflammatory response in the ischemic brain, which was accompanied by increased cerebral blood flow and reduced detachment of astrocyte endfeet from the capillary basement membrane. The RNA sequencing data showed that BLR promoted the upregulation of extracellular matrix and angiogenesis pathway-related genes; in particular, BLR significantly increased the expression of the chondroitin sulfate proteoglycan 4 (CSPG4) gene, enhanced the membrane location of CSPG4, and promoted its downstream signaling protein expression, which is associated with KDEL receptor (KDELR) activation. In addition, activated KDELR further increased the phosphorylation of mitogen-activated protein kinases after BLR treatment. Taken together, our data showed that BLR could protect against ischemic brain injury and may serve as a new promising therapeutic candidate drug for ischemic stroke, and that KDELR might act as both a sensor and effector to activate CSPG4 to increase cerebral blood flow.

## 1. Introduction

Ischemic stroke accounts for more than 80% of strokes. Vascular recanalization techniques such as drug thrombolysis and endovascular treatment have led to great progress in stroke treatment [1]. Unfortunately, although the rate of vascular recanalization has increased, the proportion of patients who benefit from the treatment remains relatively low, and this contradiction is still an important problem in clinical practice [2]. Timely restoration of blood flow to the ischemic tissue is the key to ensuring success for acute stroke therapy. The findings from numerous clinical studies suggest that ischemic stroke patients with good collateral circulation are good candidates for early reperfusion after endovascular treatment [3], but the mechanism is still unclear.

Proteoglycan is a ubiquitous surface component of eminently viable cell phenotypes and plays a role in stimulating endothelial cell motility and nerve cell maturation [4]. The neural progenitor marker neuron glia-2 (NG2), also named as chondroitin sulfate proteoglycan 4 (CSPG4), is a transmembrane integral chondroitin sulfate proteoglycan [5]. Owing to its extended extracellular domain and endocellular motif, CSPG4 has the potential to promote interactions between cells and the extracellular matrix (ECM), spanning from the sequestration of growth factors, extracellular matrix collagens, and metalloproteinases to cell surface receptors [6]. Glial cells expressing CSPG4 are abundantly present in the brain and demonstrate a reactive response to ischemic damage [7], and CSPG4 has been demonstrated to promote cell survival via regulating the interactions between brain-related cells, to stimulate neurovascular unit (NVU) function during microvascular morphogenesis, and to regulate brain blood flow [8]. Furthermore, CSPG4 is also identified as a signal transducing protein through binding to its cytoplasmic C-terminal scaffolding and signaling proteins, which accelerates integrin-mediated adhesion and activates the prosurvival signal molecules extracellular signal-regulated kinase (ERK) 1/ERK2 and phosphatidylinositol 3-kinase (PI3K)/protein kinase B (Akt) [9]. Although CSPG4 has the unique capability to affect numerous cell behaviors and cellular processes and has a wide range of ECM ligands that facilitate CSPG4 to serve as a pharmacologic mediator in controlling the neurovascular signaling network, whether CSPG4 could serve as a therapeutic target of neuroprotection in ischemic stroke has not been explored.

In recent decades, the exploration and application of traditional herb-derived components, an important source of potential neuroprotective drugs, have attracted great attention in the search for pharmacological targets for stroke treatment [10]. As a class of glycoside compounds derived from natural plants, flavonoids and flavonoid glycosides have been demonstrated to be effective in reducing neurological impairment in ischemic stroke [11]. Therefore, as part of our ongoing project focusing on discovering new neuroprotective agents from natural sources, we have recently been focusing on investigating the neuroprotective effects of flavonoids and flavonoid glycosides using *in vivo* stroke models. Linarin, a disaccharide flavonoid glycoside isolated from Chrysanthemum *indicum L*., has been considered as a promising natural product due to its diverse pharmacological activities, such as alleviating oxidative stress [12], inhibiting nuclear factor-kappaB- (NF-*κ*B-) dependent inflammatory activation [13], regulating the activity of neurotransmitters [14], and exerting anti-inflammatory actions. Moreover, linarin has been recently reported to protect against acute ischemic kidney and heart injury [15, 16]. However, whether linarin and its derivatives are neuroprotective has not yet been investigated in ischemic stroke.

In this study, using a mouse model of middle cerebral artery occlusion (MCAO), we demonstrated that 4′-benzylapigenin-7-*β*-rutinoside (BLR), a derivative of linarin, could reduce ischemic brain injury and promote the recovery of neurological deficits by improving the blood supply to postischemic brain tissue as well as the inhibition of neuronal apoptosis and inflammatory damage, and this protection was mediated by the activation of CSPG4-dependent signaling pathways due to the internalization of the endoplasmic reticulum (ER) retention signal carboxyl-terminal Lys-Asp-Glu-Leu receptor (KDELR) and mitogen-activated protein kinase (MAPK) activation.

## 2. Materials and Methods

### 2.1. Reagents

The linarin derivative BLR was synthesized and provided by Dr. GX Ma, IMPLAD of Peking Union Medical College, Beijing. The derivatization of BLR was performed according to the following procedure. The starting material (1 mmol, g) was dissolved in dichloromethane (5 ml), and subsequently, acetic anhydride (2 mmol, 0.2 ml) and triethylamine (5 mmol, 0.7 ml) were added successively. Then, the reaction was completed at room temperature. The product was dissolved in dichloromethane, and boron tribromide was added in an ice bath. Then, the reaction was quenched with 5 ml distilled water after 2 h. The product was then extracted with ethyl acetate and steam dried in the organic phase to obtain the crude product and which was finally purified by silica gel column chromatography. The product (1 mmol, g) was dissolved in DMF (5 ml) added with benzyl bromide (1.2 mmol, 200 mg) and potassium carbonate (2 mmol, 278 mg), which were added successively, and the reaction ended at room temperature. Distilled water and ethyl acetate were used for extraction, and we evaporated the organic phase to dry to obtain the crude product, which was finally purified by silica gel column chromatography. The product (1 mmol, g) was dissolved in methanol (5 ml) added with sodium methoxide solution (1 mol/l in methanol, 2 ml), and the reaction ended at room temperature, and the cationic resin was added to quench the reaction. The reaction solution was concentrated and purified by silica gel column chromatography to obtain final compound 1. TLC showed that the reaction was completely filtered, and the filter cake was washed 3 times with a small amount of toluene, combined with filtrate and vacuum distillation to obtain the powder. The light yellow powder underwent fast column separation and recrystallization with n-hexane to obtain the end product. The purified product was finally characterized by ^1^H and ^13^ C-NMr spectra through 600 M NMR, and the purity of BLR was over 98%, as determined by high performance liquid chromatography (HPLC) analysis.

### 2.2. Animal Groups

C57BL6/j male mice (8-10 weeks) were purchased from Beijing Vital River Laboratory Animal Technology (Peking, China). Mice were bred in an inverse 12 h/12 h light/dark cycle at a temperature ranging from 20°C to 23°C with 50% humidity. All procedures were executed based on the US National Institutes of Health Guide for the Care and Use of Laboratory and approved by the ethics committee of the Shenzhen Second People's Hospital (registration number: 20181106007, date: 11/06/2018). The online tool QuickCalcs (http://www.graphpad.com/quickcalcs/) was utilized to randomize animals to experimental groups, i.e., the sham, vehicle, and BLR groups. A transient focal ischemic MCAO stroke model was utilized and is described below. The neurological scores and infarct volumes were assayed by a researcher blinded to animal group assignment.

### 2.3. Focal Cerebral Ischemic Stroke Model

Transient MCAO was performed on male mice (8-10 weeks) as previously described [17]. Briefly, the mice were subjected to anesthesia induction using 5% isoflurane, thereafter to 1.0-1.5% isoflurane. Anesthesia was maintained during the whole operation, and rectal temperature was maintained at 37.0°C ± 0.2°C by a heating pad (Alcott Biotech, Shanghai, China). Regional cerebral blood flow (rCBF) of the MCA territory was measured by laser Doppler flowmetry (Moor Instruments, Devon, UK). Focal cerebral ischemia was conducted by inserting a monofilament (RWD, Shenzhen, China) into the left internal carotid artery via the common carotid artery. After 30 min of occlusion, the filament was removed, and the incision was sutured. Then, the mice were returned to their home cages and sacrificed 24 h after surgery. Mice that had intracranial hemorrhage, an rCBF reduction < 75% during MCAO, or an rCBF recovery > 70% after 10 min of reperfusion were excluded.

### 2.4. Laser Speckle Contrast Imaging

The mice of each group were anesthetized with isoflurane during the whole procedure. The hair was shaved, and the skull was exposed to the laser detector for cerebral blood flow imaging. Cerebral blood flow was scanned by the laser speckle contrast imaging (LSCI) system (RWD, Shenzhen, China), and the images and videos were processed with the RWD software according to the manufacturer's instructions. CBF was quantitated and expressed as a blood flow index that was calculated as the ratio of the region of interest (ROI) areas between the ipsilateral and contralateral hemisphere.

### 2.5. Neurological Function Evaluation

A 48-point scoring system was utilized to assess neurological deficits, as described previously [18]. This scoring system includes general status (spontaneous activity, body symmetry, and gait; 0-12), simple motor deficit (forelimb asymmetry, circling, and hind-limb placement; 0-14), complex motor deficits (vertical screen climbing and beam walking; 0-8), and sensory deficits (hind limb, trunk, vibrissae, and face touch; 0-14). The total score was calculated by adding 4 individual scores, where 0 equals to no deficit and 48 equals to maximal deficit. Moreover, rotarod and open field tests were also performed to evaluate stroke outcomes, as we described previously [19, 20]. In brief, mice were placed on an accelerating rotating rod (Med Associates Inc., St Albans, VT, USA) to perform the rotarod test, and the data were recorded. Mice were placed in an open field for testing motor ability (CleverSys Inc., Reston, VA, USA), and spontaneous motor activity was recorded for 10 min or 60 min with a 20 sec delay. The latency to fall from the rod, the distance travel, and the time spent in the center were recorded and analyzed by the EthoVision XT15 software (Noldus, Holland). All mice were pretrained for three days before MCAO. All evaluations were performed in a blinded manner.

### 2.6. Infarct Volume

Mice were killed at 24 h post stroke. The brain slices were harvested and stained with 2,3,5-triphenyltetrazolium chloride (TTC) (Sigma, St. Louis, MO, USA). TTC is enzymatically reduced by dehydrogenase to generate red staining in the noninfarcted brain tissue area, and in contrast, pale staining corresponds to infarct areas. In brief, the infarct area of each 1 mm thick section was measured by subtracting the noninfarcted area in the ipsilateral hemisphere from the total area of the contralateral hemisphere. Then, the final infarct volume was calculated by summing the infarct areas in all sections and multiplying by the section thickness.

### 2.7. Brain Clearing

The CUBIC (clear, unobstructed brain/body imaging cocktails) protocol was used to stain whole-brain cerebromicrovessels, according to published protocol [21]. Briefly, the brains were sequentially perfused with saline and 4% PFA followed by FITC-albumin, which was used for staining brain microvessels. Thereafter, the brain samples were washed with PBS containing 0.01% sodium azide before incubation at 37°C overnight with 20 ml semidiluted CUBIC-L composed of a mixture of 25% urea, 25% Quadrol, and 15% Triton X-100 (Sigma, St. Louis, MO, USA) with gentle shaking. The mixture was refreshed every 1-2 days for 15 days. Thereafter, the brains were incubated at room temperature overnight with half-diluted CUBIC-R, consisting of a mixture of 25% urea, 50% sucrose, and 10% triethanolamine (Sigma, St. Louis, MO, USA) with gentle shaking. The mixture was replaced every 1-2 days until the brain turned into transparent. The transparent brains were imaged under an ultramicroscope (LaVision BioTec).

### 2.8. Terminal Deoxynucleotidyl Transferase dUTP Nick End Labeling (TUNEL)

Cell apoptosis was detected by TUNEL staining using an in situ apoptosis detection kit (Millipore, MA, USA) based on the manufacturer's instructions. In brief, the brain slices were incubated with proteinase K (20 *μ*g/ml) at room temperature for 15 min and then incubated with TdT enzyme in a humidified chamber at 37°C for 1 h. The brain tissues were incubated with working-strength antidigoxigenin conjugated at room temperature for 30 min after washing with PBS. The images of TUNEL staining were acquired and observed using a fluorescence microscope (Leica, Germany).

### 2.9. RNA Sequencing and Bioinformatics Analysis

RNA sequencing was performed according to previously reported methods [22]. Total RNA was isolated from brain tissue using TRIzol reagent (Invitrogen, Carlsbad, Calif., USA) and then purified with an RNeasy Mini Kit (Qiagen, Hilden, Germany) according to the manufacturer's instructions. Then, the RNA integrity number was examined by an Agilent Bioanalyzer 2100 (Agilent Technologies, Santa Clara, CA, USA) and quantified using Qubit (Thermo Fisher). Oligo (dT) was used twice for selection with Dynal magnetic beads (Invitrogen), and poly-A RNA was fragmented before cDNA synthesis. After adapter ligation, the cDNA libraries were amplified and purified with magnetic beads and then validated using an Agilent 2100 Bioanalyzer. The products were quantified and sequenced on an Illumina X-ten platform using SBS v3 reagents. Sequence filtering, sequence alignment, and transcript quantification were realized by Fastp, hisat2, and feature counts, respectively. Differentially expressed genes were analyzed using the DESeq package in R, and the differentially expressed genes were classified depending on whether they were up- or downregulated with more than a 1.5-fold change and *P* < 0.05. GO and pathway analyses were performed using the clusterProfiler package software in R. Then, Fisher's exact test was performed to choose the significant GO terms and pathways, and *P* < 0.05 was identified as the threshold of significance. For the construction of the interaction network, all differential proteins were imported into the STRING database to obtain the relationship between genes and to display the relationship list between the proteins with the software Gephi, which was used to perform PageRank and modularity class analysis. Heatmaps were constructed according to the most clustered genes in the interaction cluster.

### 2.10. Immunoblotting Assay

The cortical tissues of MCAO mice were dissected for western blotting. Briefly, the protein was extracted by RIPA buffer containing protease inhibitor cocktail, phosphatase inhibitor cocktail, and phenylmethanesulfonyl fluoride (Roche, IN, USA). The concentration of protein was examined using BCA kits. Equal amounts of protein were separated by electrophoresis in 10% sodium dodecyl sulfate polyacrylamide gels and were transferred to nitrocellulose membranes which were incubated with 5% nonfat milk solute that contained 0.1% Tween-20 (TBST) for 2 h. Then, the membranes were incubated with the following primary antibodies at 4°C overnight: anti-CSPG4, anti-KDELR, anti-PMCA1, anti-phospho-Akt and total Akt, anti-phospho-Src and total Src, anti-phospho-p38 and total-p38, anti-phospho-CREB and total-CREB, anti-GRP94, anti-GRP78, and anti-*β*-actin (Abcam, Cambridge, USA). Membranes were then incubated for 1 h with the secondary antibodies, which were purchased from Abcam (Abcam, Cambridge, USA). These blots were visualized by a gel imager (Bio-Rad, USA).

### 2.11. Immunofluorescence

For immunofluorescence analysis, the mice were transcardially perfused with 0.9% saline and then perfused with ice-cold 4% paraformaldehyde. Excised brains were incubated in 4% paraformaldehyde overnight. Sections (2 *μ*m thick) of the brain were cut on a freezing microtome (Leica, Germany). The brain slices were subjected to immunofluorescence to detect Iba-1 (antibody information) expression levels and double immunostaining for collagen IV and GFAP (antibody information). Briefly, the brain slices were permeabilized with 0.1% Triton X-100 for 1 h and blocked in blocking buffer containing serum for 30 min. Then, the slices were incubated at 4°C overnight with antibodies against Iba-1, collagen IV, and GFAP. After rinsing with PBS, the primary antibodies were detected using FITC- or Cy5-conjugated goat anti-rabbit or anti-mouse antibodies for 1 h at room temperature. The brain slices were then incubated with DAPI (0.1 *μ*g/ml) for 15 min. Images of immunostaining were obtained using a Leica confocal laser scanning microscope (Leica, Germany).

### 2.12. Immunoprecipitation

Co-IP was used to detect the interaction between KDELR and CSPG4. Briefly, the cortical tissue was incubated with ice-cold IP lysis buffer for 10 min. The tissue lysates were centrifuged at 12,000 rpm for 10 min at 4°C, and the supernatant was collected and then precleared with control agarose resin. A total of 1 mg of protein was incubated with 10 *μ*g of antibody immobilization AminoLink Plus coupling resin on a rotator overnight at 4°C. Immunoprecipitates were washed four times with lysis buffer, and proteins were eluted by elution buffer. Then, the sample was analyzed by immunoblotting.

### 2.13. Enzyme-Linked Immunosorbent Assay

Mice were perfused with 0.9% saline (pH 7.4), and the brains were removed rapidly. The cortical samples were weighed and homogenized with PBS. The levels of ICAM-1, TNF-*α*, IL-1*β*, and IL-6 were determined by ELISA kits (Nanjing Jiancheng Bio, China).

### 2.14. Purification of Subcellular Fractionation

After the last administration, tissue proteins were gently resuspended in cytoplasmic buffer (10 mM HEPES, 0.5 mM DTT, 1.5 mM MgCl_2_, 10 mM Na_2_MoO_4_, 10 mM KCl, 25 mM NaF, 20 mg/ml aprotinin, 0.1% NP-40, 2 mM PMSF, and 0.2 mM Na_3_VO_4_) and were centrifuged at 900 × *g* for 10 min. The nuclear pellets were prepared as described below. The cytoplasmic supernatant was recentrifuged at 900 g, and the acquired supernatant was further centrifuged at 1000 × *g* for 30 min. Then, the cytosolic supernatant was subjected to immunoblotting. In addition, different subcellular fractions were separated using endoplasmic reticulum extraction kit (Bjbalb, China) and Golgi body extraction kit (Bjbalb, China). These fractions were broken with a cell disruptor (Scientz Ultrasonic Homogenizer, China).

### 2.15. Statistical Analysis

The results are presented as the mean ± standard deviation (SD). Statistical analyses were executed with the SPSS 17.0 software in this study. Neurological scores and infarct volumes were analyzed by two-way ANONA followed by Bonferroni post hoc testing. Statistically significant differences were considered at *P* < 0.05.

## 3. Results

### 3.1. Preliminary Study and Synthesis of the Linarin Derivative BLR

**Figure 1:**
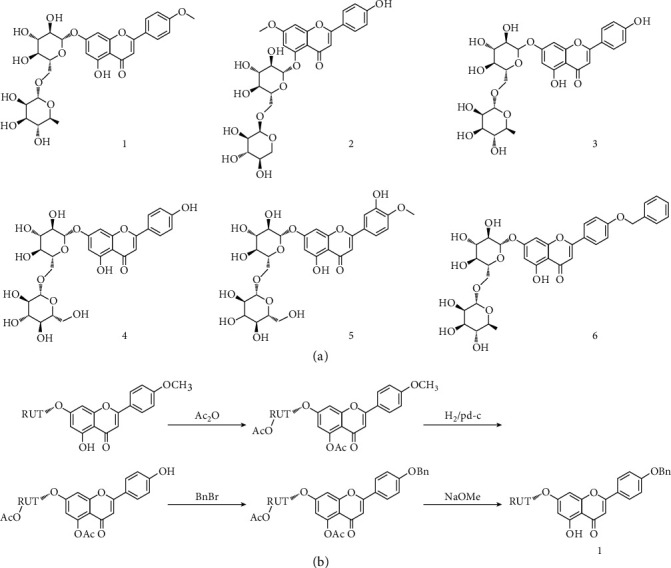
Preliminary and synthesis of a linarin derivative 4′-benzylapigenin-7 -*β*-rutinoside (BLR). (a) The names and structures of linarin and its derivates and (b) the synthetic route and chemical structure of BLR from linarin. The neuroprotective effects of linarin and its derivatives were evaluated by the mouse MCAO model, and the result of preliminary study was not shown.

In a preliminary study, we screened the neuroprotective effect of linarin and its derivatives (Figure 1(a)) in a mouse MCAO model and found that the derivative BLR showed the best neuroprotection (data not shown). Therefore, we chose to study the neuroprotective effect of BLR. To achieve a sufficient amount of BLR for this study, we synthesis BLR through a four-step reaction, and the synthetic steps and the chemical structure of BLR are shown in Figure 1(b) with a yield of 78%. Compound BLR: ^1^H-NMR (MeOD-*d*_4_) *δ*_H_:7.18(1H, d, *J* =2.4 Hz, H-1), 6.68(1H,dd, *J* =2.4 Hz, H-3), 6.30(1 H, s, H-7), 7.65(2 H, d, *J* =8.4Hz, H-10, 15), 6.88(2 H, d, *J* =8.4 Hz, H-12, 13),7.48(2 H, m, H-18,22), 7.40(2 H, m, H-19, 21),7.32(1 H, m, H-20), 5.07(1 H, d, *J* =4.2 Hz, H-1'), 4.20(1 H, m, H-2'), 3.68(1 H, m, H-3'), 3.60(1 H, m, H-4'), 3.78(1 H, m, H-5'), 3.61(1 H, m, H*α*-6', 3.36(1 H, m, H*β*-6'), 5.40(1 H, m, H-7'), 3.90(1 H, m, H-8'), 3.70(1 H, m, H-9'), 3.60(1 H, m, H-10'), 3.71(1 H, m, H-11'), 1.08(3 H, d, *J* =6.0 Hz, 11'-CH_3_). ^13^C-NMR (MeOD-*d*_4_) *δ*_C_: 118.1(C-1), 159.5(C-2), 125.2(C-3), 145.7(C-4), 124.0(C-5), 178.4(C-6), 107.6(C-7), 163.2(C-8), 156.3(C-9), 131.8(C-10), 126.3(C-11), 129.0(C-12), 131.0(C-13), 128.1(C-14), 126.3(C-15), 70.8(C-16), 136.7(C-17), 127.1(C-18,22), 128.8(C-19,21), 127.6(C-22), 105.6(C-1'), 75.4(C-2'), 78.9(C-3'), 71.8(C-4'), 80.8(C-5'), 68.6(C-6'), 112.0(C-7'), 73.8(C-8'), 72.4(C-9'), 73.7(C-10'), 74.2(C-11'), 17.0(11'-CH_3_).

### 3.2. BLR Treatment Reduced the Infarction Volume and Improved Neurological Function after MCAO *In Vivo*

**Figure 2:**
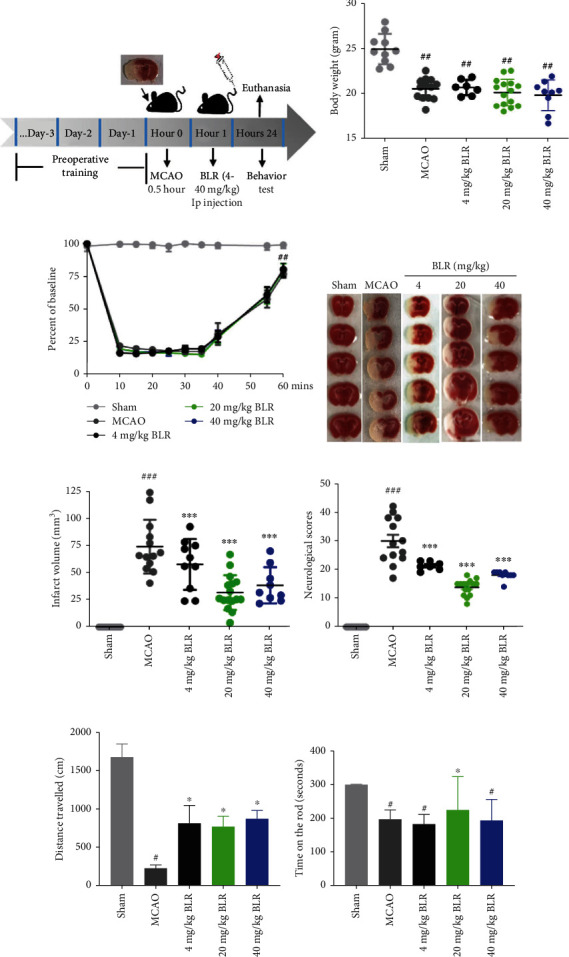
Delivery of BLR improved short-term stroke outcomes. The mice were subjected to 30min MCAO and 24h reperfusion and treated with BLR at the dose of 4, 20, and 40mg/kg or vehicle via intraperitoneal at 1h after reperfusion onset; the series of battery behavior tests were performed 24h post stroke. (a) Schematic diagram of the experimental design. (b) Body weight was monitored over time. ^##^*P* < 0.01 compared with the sham group (*n* = 10 per group). (c) LDF recorded the CBF changes in ischemic core of the MCA territory (*n* = 10 per group). ^##^*P* < 0.01 compared with the sham group. (d) Representative TTC-stained images of brain slices. (e) Infarct volume of TTC-stained brain slices was quantitated (*n* = 10 per group). ^###^*P* < 0.001 compared with the sham group. ^∗∗∗^*P* < 0.01 versus MCAO group. (f) Neurological defects were evaluated by neurologic scores (*n* = 10 per group). ^###^*P* < 0.001 compared with the sham group. ^∗∗∗^*P* < 0.001 versus MCAO group. (g) Open field and (h) rotarod tests were evaluated presurgery (day 0) at baselines and at 24 hours after MCAO (*n* = 10 per group). Data were analyzed by two-way ANOVA, followed by two-tailed unpaired *t*-tests. ^#^*P* < 0.05 compared with the sham group. ^∗^*P* < 0.05 versus MCAO group.

To evaluate the neuroprotective effect of BLR, mice were subjected to 30 min occlusion; thereafter, BLR was administered by intraperitoneal (IP) injection 1 h after reperfusion onset. The mice were sacrificed 24 h post stroke. The experimental process is shown in Figure 2(a). As shown in Figure 2(b), MCAO treatment significantly induced weight loss in the MCAO and all BLR-treated groups (4 mg/kg, 20 mg/kg, and 40 mg/kg) compared to the sham group. Laser Doppler (Moor, UK) assessment showed that the CBF dropped to 20% and flowed back to approximately 80% of the preischemic level in the MCA territory of the ischemic hemisphere after the insertion and removal of the filament, which confirmed the success of MCAO surgery (Figure 2(c)). The results of TTC staining showed that MCAO insult significantly increased the infarction volume when compared with the sham group, and the administration of 4, 20, or 40 mg/kg significantly reduced the infarction volume of MCAO mice from ~75 mm^3^ to ~60 mm^3^, ~35 mm^3^, or ~40 mm^3^, respectively (Figures 2(d) and 2(e)), and the BLR 20 mg/kg dose group had the most obvious effect. Moreover, all BLR-treated group mice showed a significantly improved neurological scores, compared to the vehicle group (MCAO group) (Figure 2(f)). Short-term behavioral outcome assessment showed that compared with vehicle mice, mice treated with BLR at a dose of 20 mg/kg traveled further in the open field test (Figure 2(g)) and consistently performed better on the rotarod test (Figure 2(h)) at 24 hours after MCAO. In view of the most obvious neuroprotective effect of the 20 mg/kg BLR group, we chose this dose for subsequent experiments. Taken together, these data suggested that postischemic administration of BLR was neuroprotective in a mouse model of MCAO.

### 3.3. BLR Treatment Reduced Cell Apoptosis and Inflammatory Responses in Ischemic Brain Tissue

**Figure 3:**
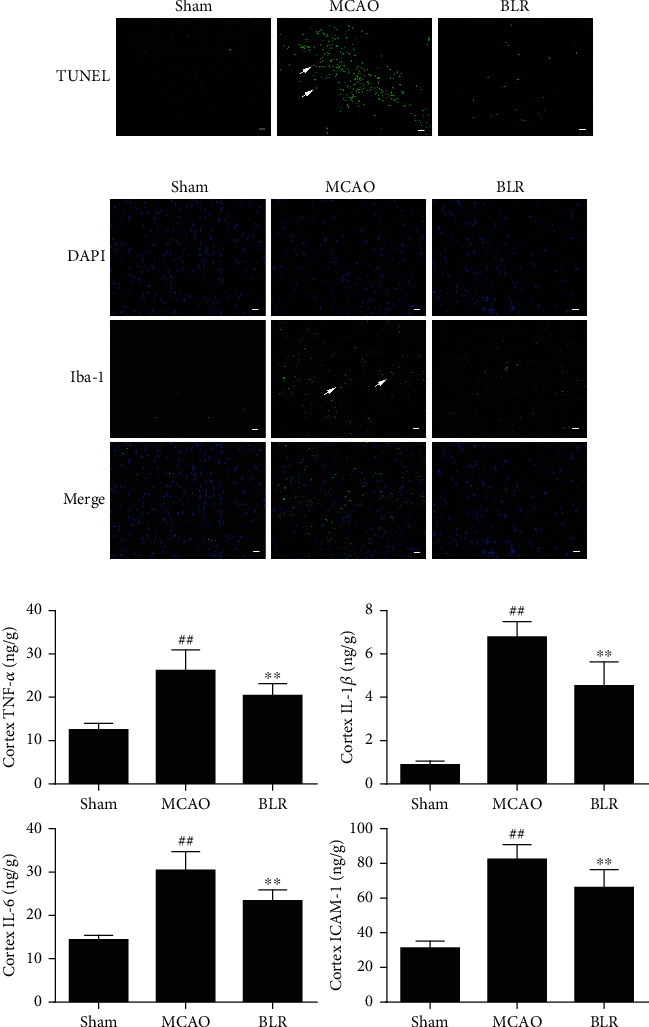
BLR attenuated MCAO-induced apoptosis and inflammation in mice. (a) The images of apoptotic cells in brain slices assessed by TUNEL assay (*n* = 6 for each group). White arrows represent TUNEL-positive cells, scale bar = 25 *μ*m. (b) Immunofluorescence images of Iba-1 (*n* = 6 per group). Scale bar = 25 *μ*m. (c) The levels of inflammatory cytokines including TNF-*α*, IL-1*β*, IL-6, and ICAM-1 were detected by ELISA (*n* = 6 per group). ^##^*P* < 0.01 compared with the sham group. ^∗∗^*P* < 0.01 compared with MCAO group.

Next, we tested whether the effect of BLR neuroprotection was achieved by reducing MCAO-induced cellular apoptosis and inflammation. As shown in Figure 3(a), the mice subjected to MCAO showed an increased number of TUNEL-positive cells in the ischemic cortex compared with the sham group, and this increase was significantly blocked by BLR treatment. We also studied the effects of BLR on microglial activation and inflammation in the ischemic brain. As shown in Figure 3(b), the immunostaining of Iba-1 (a biomarker of microglia) was very weak in the cortex of sham-operated mice, and compared with the sham group, a significant increase in the number of Iba-1-stained cells was observed after MCAO, and this increase was significantly suppressed by BLR treatment. Similarly, MCAO resulted in a marked increase in the production of TNF-*α*, IL-1*β*, IL-6, and ICAM-1 in ischemic brain tissue, which was also significantly inhibited by BLR treatment (Figure 3(c)). These data indicated that postischemic administration of BLR could effectively inhibit cellular apoptosis and inflammation in the ischemic brain tissue.

### 3.4. BLR Promoted the Restoration of Blood Flow to Ischemic Brain Tissue

**Figure 4:**
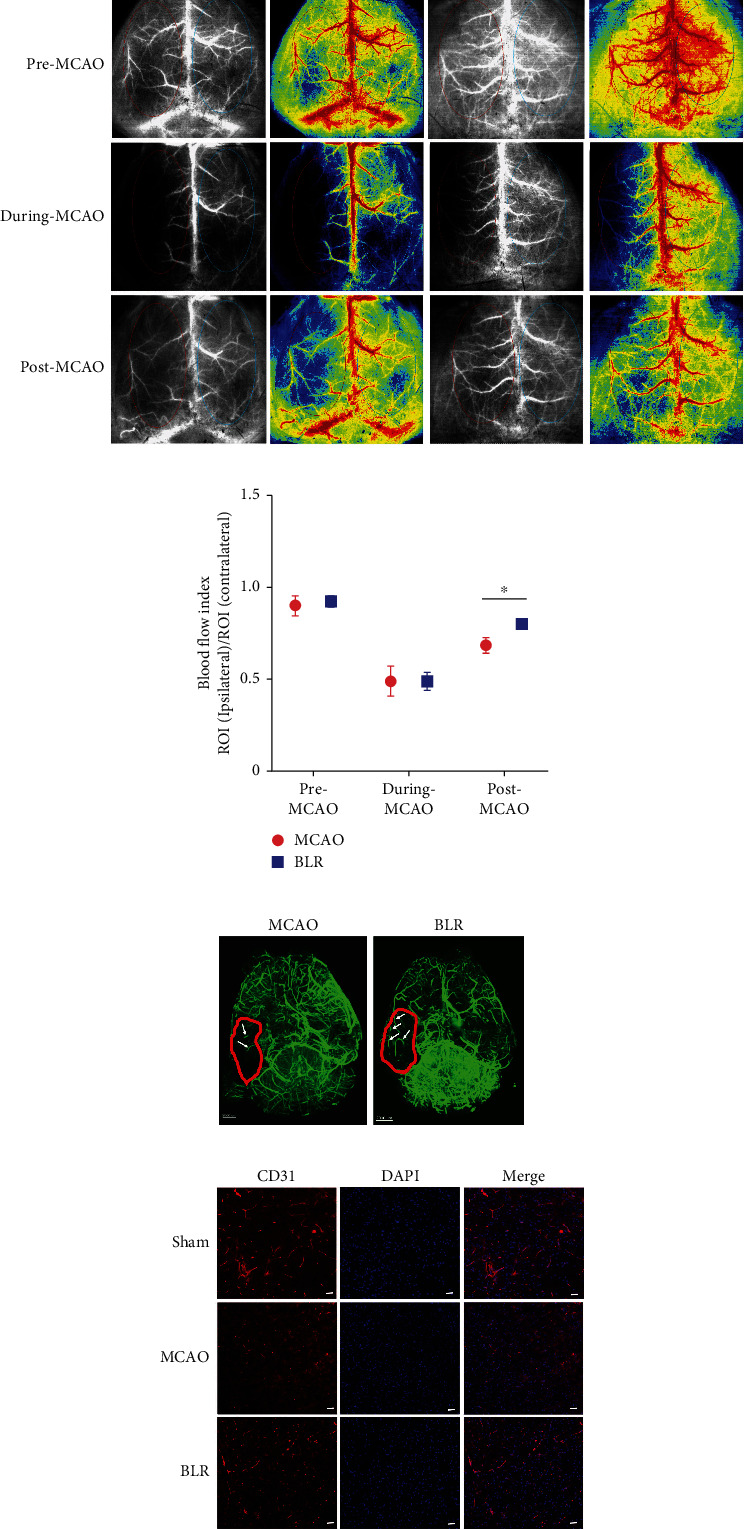
BLR inhibited cerebral blood flow decline in ischemic zone induced by MCAO. (a) Cerebral blood flow was detected by LSCI in entire MCAO process (including pre-MCAO, during MCAO, and post-MCAO) (*n* = 6 per group). Representative laser speckle contrast images of surface cortical CBF. Red oval represents ROI of ipsilateral hemisphere; green oval represents ROI area of contralateral. (b) Blood flow index changes (lpsi ROI/control ROI) of CBF in MCAO or BLR treatment (*n* = 6 per group). ^∗^*P* < 0.05 compared with the MCAO group. (c) 3D volume rendering of the vascular architecture in ischemic stroke. Brain transparent was realized by cubic clarity in the mouse MCAO model. The brain of the mice was clarified by CUBIC (*n* = 6 per group) after MCAO and BLR treatment. The ischemic regions labeling with red circle were detected by ultramicroscope (*n* = 6 per group). White arrows point to the areas which represent the blood vessels. Scale bar = 2000 *μ*m. (d) Representative immunofluorescence images of CD31 (*n* = 6 per group) to evaluate the capillary density in ischemic brain tissue. Scale bar = 25 *μ*m.

To evaluate the effect of BLR on cerebral blood supply and cerebrovascular reactivity after MCAO, we monitored the changes in CBF before MCAO (pre-MCAO), during MCAO, and after reperfusion (post-MCAO) using a LSCI system, and the blood flow index was used as a quantitative parameter of CBF. Our results showed that MCAO led to a significant reduction in CBF for both the vehicle group and the BLR-treated group compared with their pre-MCAO CBF levels (blood flow index: 0.49 ± 0.08 vs. 0.49 ± 0.05). Notably, there appeared to be more CBF perfused back to ischemic brain tissue following reperfusion in the BLR-treated group than in the vehicle-treated group (blood flow index: 0.68 ± 0.04 vs. 0.80 ± 0.03) (Figures 4(a) and 4(b)). As LSCI can monitor CBF only along the brain surface, we established the CUBIC method and investigated the effect of BLR on the distribution and opening of cerebromicrovessels in three-dimensional structures (i.e., the transparent brain). As anticipated, there were more surviving endothelial cells that were stained with FITC-albumin in the infarct rim of the BLR-treated mice than in the vehicle-treated mice (Figure 4(c)). The 3D reconstruction clearly showed that there were more cerebral microvessels in the BLR-treated mice than in the vehicle-treated mice, and CD31 staining also showed a significant reduction in cerebromicrovascular density in MCAO mice compared with sham-operated mice, while BLR treatment significantly reversed this reduction (Figure 4(d)). These data demonstrated that BLR treatment could improve blood supply to the ischemic brain tissue during reperfusion, and this effect might be due to BLR-induced better compensatory responsiveness of peri-infarct microvessels.

### 3.5. BLR Reduced the Segregation of Astrocyte Endfeet from the Capillary Basement Membrane of the NVU Induced by MCAO

**Figure 5:**
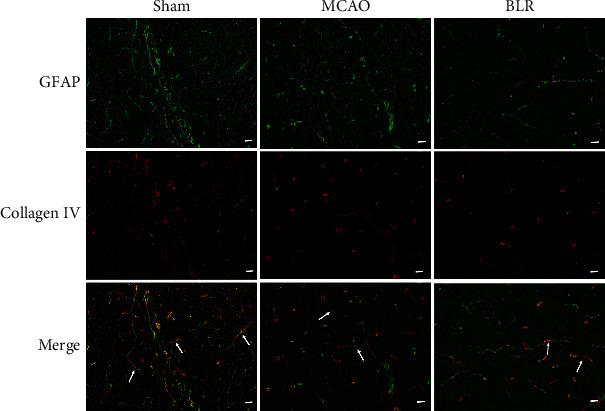
BLR treatment reduced disconnection of the astrocyte endfeet from the capillary basement membrane after ischemic stroke. Double immunofluorescence images of GFAP and collagen IV (*n* = 6 per group). Arrowheads indicate to the detachment degree of the GFAP-positive astrocyte endfeet from the collagen IV-positive basement membrane. Scale bar = 25 *μ*m.

Neurovascular coupling can lead to a compensatory increase in local CBF to meet the increased energy needs of specific brain regions [23]. To further determine how BLR ameliorated CBF in MCAO mice, we examined the effect of BLR treatment on neurovascular coupling by checking the integrity of the NVU using double immunostaining of collagen IV (representing brain capillary basement membrane) and GFAP (perivascular astrocytes). As shown in Figure 5, MCAO significantly induced the segregation of GFAP-positive astrocyte endfeet from the collagen IV-positive basement membrane in comparison with that in the sham group. Of note, BLR treatment significantly decreased the dissociation of astrocyte endfeet processes from the basal lamina in ischemic brain microvessels. These data indicated that BLR significantly reduced the segmentation of perivascular astrocytes from brain capillaries in the ischemic brain region and might thus ameliorate MCAO-induced disruption of neurovascular coupling.

### 3.6. BLR Treatment Augmented the Gene Expression Associated with the ECM and the Angiogenesis Pathway after Cerebral Ischemia

**Figure 6:**
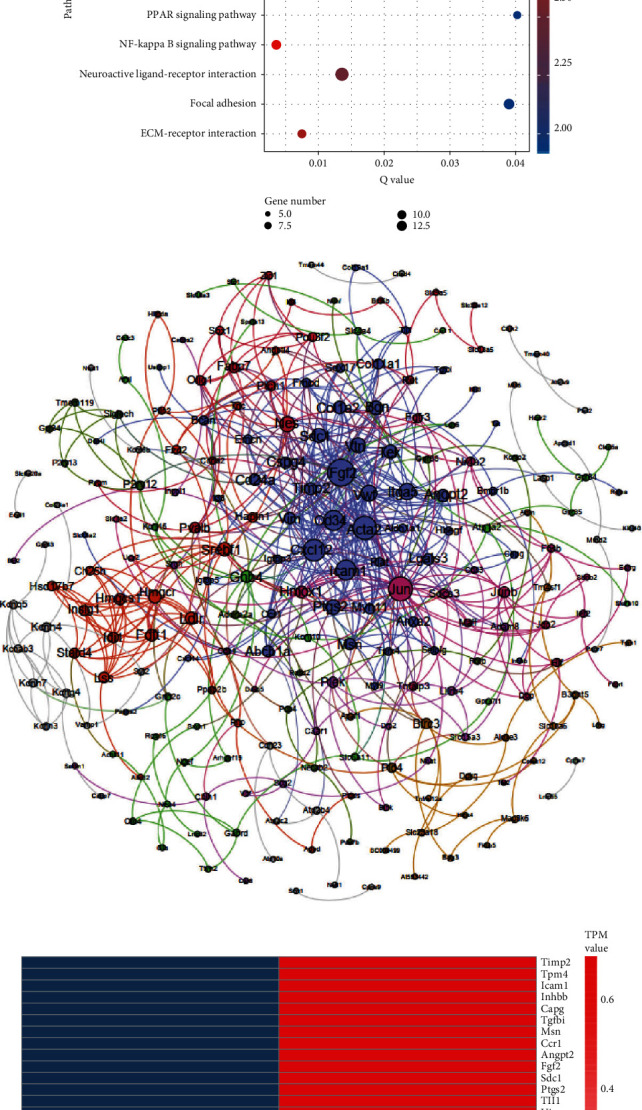
Effects of BLR on the vasculature and angiogenesis pathway-related gene expression by RNA sequencing analysis. (a) Scatter plot showed the differences of gene expression between MCAO group and BLR group. Red dots represented as upregulated genes, blue dots represented as downregulated genes, and gray dots indicated no change. (b) Pathway enrichment analyses were performed on the clusterProfiler software using ClueGO module at 0.05 significant level. (c) Interaction network and cluster relationship of differentially expressed genes involved in MCAO mice treated with BLR or saline were shown. (d) Heatmap revealing RNA sequencing result of upregulation and downregulation of vasculature and angiogenesis pathway-related gene expression between MCAO group and BLR group (*n* = 3 per group). Red blocks indicated high expression, and blue blocks indicated low expression.

To further study the mechanism of the BLR-afforded neurovascular protective effects in MCAO mice, we analyzed the effect of BLR on the gene expression profile of ischemic brain tissue using the Illumina X-ten platform. After normalization, we identified 162 upregulated and 133 downregulated genes (|log2(fold change)| ≥ 1 and *P* < 0.05) (Figure 6(a)). To characterize the predominant pathways, we performed KEGG pathway enrichment analysis and found that a large number of regulatory pathways and genes were enriched in the BLR-treated group, particularly ECM-receptor interactions, the NF-*κ*B signaling pathway, and other pathways favorable for cell survival (Figure 6(b)). Based on the correlation among genes, we performed a gene-gene interaction network assay to identify the important genes that are associated with BLR's neurovascular protection against ischemic injury. The results indicated that the most clustered genes were ECM and angiogenesis pathway-related genes (Figure 6(c)). Among them, twenty-eight genes were upregulated in MCAO mice subjected to BLR treatment, and of note, the difference in CSPG4 expression was the most significant (Figure 6(d)).

### 3.7. KDELR-Mediated Transportation and Membrane Localization of CSPG4 Contributed to the Neuroprotection of BLR

**Figure 7:**
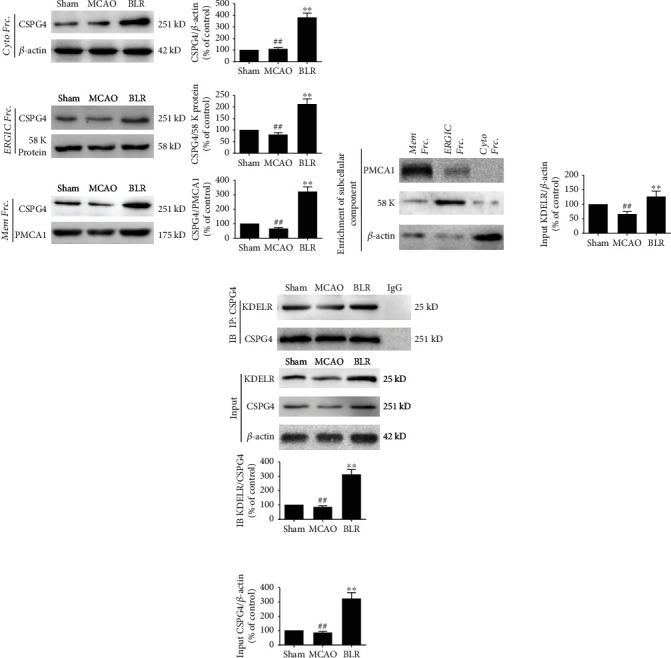
BLR induced transport and membrane localization of CSPG4 mediated by KDELR after MCAO. (a) Effects of BLR on the expression of CSPG4 in different subcellular fraction (cytoplasm, ERGIC, and membrane). (b) There is no contamination among subcellular fraction (cytoplasm, ERGIC, and membrane). (c) Effects of BLR on the expression of KDELR and CSPG4, together with the combined of KDELR to CSPG4 measured with Co-IP assays. The results of three independent experiments were termed as the mean ± SD. ^##^*P* < 0.01 compared with the sham group. ^∗∗^*P* < 0.01 compared with the MCAO group. *n* = 6 per group.

CSPG4 is a marker of oligodendrocyte progenitor cells and regulates various cell functions, such as proliferation, survival, and ECM interactions [24]. Moreover, since CSPG4 is a membrane proteoglycan, its intracellular transportation and distribution are crucial influencing factors in activating CSPG4-dependent signaling pathways [25]. To determine the effect of BLR treatment on CSPG4 activity, we analyzed the protein levels of CSPG4 in different subcellular fractions including the cytoplasm, ER-Golgi intermediate compartment (ERGIC), and membrane by western blotting and found that BLR not only elevated the total CSPG4 protein level but also significantly increased its levels in both ERGIC and membranous extracts significantly (Figure 7(a)), indicating that BLR enhanced the distribution of CSPG4 to the cytosol and the membrane. To explore the mechanism mediating the intracellular distribution of CSPG4, we examined whether the ERGIC-dependent secretory pathway and KDELR protein were involved in the vesicular trafficking of CSPG4. The interaction between KDELR and CSPG4 was detected by Co-IP. As shown in Figure 7(b), MCAO significantly reduced the amount of KDELR coimmunoprecipitated by CSPG4 and suppressed the expression level of KDELR. However, BLR treatment increased KDELR/CSPG4 complex formation and upregulated the protein levels of both KDELR and CSPG4 (Figure 7(c)). These results suggested that BLR might reinforce the membrane localization and intracellular transport of CSPG4 by activating KDELR under ischemic conditions.

### 3.8. BLR Stimulated the CSPG4 Pathway and KDELR-Associated MAPK Cascades after Ischemic Stroke

**Figure 8:**
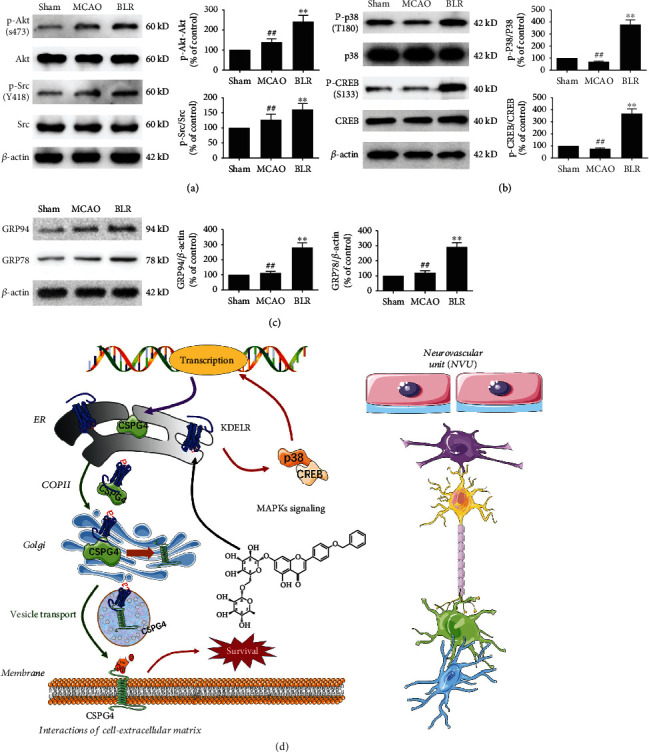
BLR both aroused CSPG4 pathway and KDELR-associated MAPK cascades in ischemic stroke. (a) Effects of BLR on the phosphorylation levels of CSPG4 downstream targets (phosphorylated Akt and phosphorylated Src). (b) Effects of BLR on the phosphorylation levels of KDELR-associated MAPK cascades (p38 and CREB). (c) BLR affected the expression levels of ER-resident molecular chaperones including GRP94 and GRP78. (d) A schematic diagram in which a derivate of linarin (BLR) protects against neurovascular unit injury via KDELR-dependent CSPG4 activation in ischemic stroke (*n* = 6 per group). ^##^*P* < 0.01 compared with the sham group. ^∗∗^*P* < 0.01 compared with the MCAO group.

In addition to KDEL-mediated enhancement of CSPG4 membrane localization, since elevated CSPG4 has also been involved in cell proliferation, survival, and ECM interactions by activating the Akt and Src pathways [24], we next evaluated the effects of BLR treatment on CSPG4's downstream targets under ischemic stroke. As shown in Figure 8(a), the protein expression of phosphorylated Akt and phosphorylated Src, two downstream molecules in the CSPG4 pathway, was increased dramatically after exposure to BLR, as was the increase in CSPG4 protein levels. To elucidate the mechanisms of BLR treatment in regulating CSPG4, we also focused on the diversiform function of KDELR. Apart from protein quality control, KDELR is also a seven-transmembrane domain protein coupled to heterotrimeric G proteins; thus, KDELR simultaneously participates in the regulation of certain signal transduction pathways, such as MAPK signaling cascades. In recent studies, the activation of p38/MAPKs has been demonstrated to promote the transcription of CSPG4 [26]; therefore, we detected the effect of BLR on the activation of the MAPK pathway and the protein expression of ER-resident chaperones. As shown in Figures 8(b) and 8(c), we found through western blotting analysis that BLR upregulated the phosphorylation levels of p38 and CREB and further increased the expression levels of GRP94 and GRP78. Interestingly, although MCAO enhanced GRP94 and GRP78, the levels of KDELR, phosphorylated p38, and phosphorylated CREB were not restored, which implied that BLR might activate KDELR in a chaperone-independent manner. Given the present results, these observations implied the positive synergetic effects of BLR-induced CSPG4 signaling activation via KDELR (Figure 8(d)).

## 4. Discussion

In the present study, we synthesized a disaccharide flavonoid glycoside to derivate BLR from linarin and investigated its protective effects on neurovascular damage in a mouse model of ischemic stroke. The major findings were as follows: (1) postischemic administration of BLR significantly reduced infarct volume, ameliorated neurological deficits, and suppressed brain cell apoptosis and inflammatory reactions during reperfusion; (2) BLR-afforded neurovascular protection might be associated with improved blood supply to ischemic brain tissue and attributed to the activation of CSPG4-dependent signaling pathways; and (3) the posttranslational modifications and vesicular transport of CSPG4 from ERGIC to the cellular membrane mediated by KDELR seemed to be a significant issue in BLR-induced neuroprotection. In addition, BLR might increase the expression of CSPG4 via the MAPK cascade-dependent positive synergetic enhancement via motivating KDELR.

Emerging evidence has shown that effective compensatory regulation of CBF is an important way to protect against ischemic brain injury, and targeting the dysfunction of CBF regulation has been an important research focus in ischemic stroke [27]. Flavonoids and flavonoid glycosides derived from natural products have been shown to provide protection against cerebral ischemia injury by promoting autophagy [28] and decreasing oxidative stress or anti-inflammation [29]. Linarin is a disaccharide flavonoid glycoside and has been reported to protect against acute ischemic heart and kidney injury [16, 30], and this protective effect may be due to its role in microcirculatory regulation [31]. However, whether linarin can regulate cerebral microcirculation to protect the brain against ischemic brain injury remains unclear. We recently synthesized a linarin's derivative named BLR, which exerted its neuroprotective function by decreasing neuronal apoptosis and inflammation accompanied by an increase in blood flow under cerebral ischemic conditions. Beneficial effects of the flavonoid fraction on microcirculation regulation have been reported, which may explain its inhibitory action on the inflammatory cascade, its protection against hypoxia-induced injury in endothelial cells, and its regulation of venous tone [32, 33]. Based on the evaluation of the effective perfusion of cerebral microcirculation, we first speculated that the role of BLR in promoting blood flow recovery was associated with the enhancement of the increase in CBF restoration in brain tissue by promoting neurovascular coupling under ischemic stroke conditions in addition to anti-inflammation, however, which cell types (especially brain microvascular endothelial cells) are responsible for BLR exerting neurovascular protection efficacy in cell models needs to be validated in future studies.

Using RNA sequencing, we found that BLR treatment induced the clustering of vasculature and ECM-receptor interaction pathway-related genes. Moreover, our data showed that BLR treatment promoted CBF restoration was accompanied by enhanced membrane localization of CSPG4. CSPG4 has been reported to act as a marker for developing cerebral vasculature [34] and has been shown to contribute to blood flow control within the capillary bed under physiological conditions [35, 36]. Similar to any other membrane-associated proteoglycan, the physiological function of CSPG4 depends on appropriate folding, precise posttranslational modifications, and correct intracellular protein transport [37]. To integrate the multitude of molecular interactions in the ECM, newly synthesized CSPG4 is initially incorporated into the endoplasmic reticulum (ER) and transported to the ERGIC-dependent secretory pathway [38]. Therefore, the intracellular transport of CSPG4 from ERGIC to the cellular membrane plays a crucial role in the maturation and activation of CSPG4. Here, our findings indicated that the activation of the KDELR protein appeared to be involved in vesicular trafficking of coat protein II-mediated CSPG4 in ischemic stroke.

KDELR has been identified as a seven-transmembrane domain protein and is coupled to heterotrimeric G proteins. Apart from participating in protein quality control, KDELR activation also mediates ER retrieval and membrane trafficking. Emerging evidence has revealed that KDELR is involved in signal transduction and thus regulates numerous important pathophysiological functions [39]. KDELR was originally demonstrated to be essential for the retrieval of ER-resident molecular chaperones, including a KDEL sequence from the ERGIC complex when the chaperones are released from the ER [40, 41]. Nevertheless, it has been proven that KDELR activation mediates bidirectional transport between the ER and the Golgi complex and from the ERGIC to the cellular membrane and external secretion, which warrants the transportation of specific substrates to their suitable locations [42, 43]. Consequently, the KDELR-mediated transport system is devoted to the dynamic equilibrium and functional control of many membranal and external proteins. Our results indicated that BLR treatment might promote posttranslational modifications, intracellular transport, and membrane localization of CSPG4 via KDELR. Furthermore, our data also demonstrated that BLR could induce the activation of KDELR-associated MAPK signaling cascades. Since p38/MAPK have been acknowledged to affect the expression level of CSPG4 [44], the result raises the possibility that BLR activates CSPG4-dependent signaling by regulating KDELR, which on the one hand enhances the membrane transport and localization of CSPG4 and, on the other hand, increases the protein level of CSPG4 by activating MAPK cascades, thus producing a positive synergetic effect in improving blood supply to ischemic brain tissue during reperfusion. It is worth pointing out that we did not perform experiments to study the effect of BLR on the KDELR pathway under normal conditions. Moreover, whether and how KDELR regulates the subcellular distribution of CSPG4 remains to be further clarified by mechanistic experiments in the future.

To the best of our knowledge, this is the first study to systemically evaluate the protective effect of BLR on ischemic stroke. Our data show that BLR treatment is effective in reducing ischemic brain injury, and this protection is attributed to BLR's action on neurovascular damage, in which BLR improves postischemic reperfusion of the ischemic brain tissue through KDELR-dependent CSPG4 activation. Moreover, our data also provide novel evidence that intracellular transportation of KDELR might be a potential stimulator for the activation of the transmembrane proteoglycan CSPG4 and ECM signaling. Another interesting assumption is that the way that KDELR modulates the CSPG4 pathway shown here may also contribute to other pharmacological actions, suggesting that KDELR may act as both a sensor and an effector to participate in many biological processes. Last, although our data support BLR as a promising therapeutic candidate drug for ischemic stroke, the mechanisms underlying BLR's neuroprotection need to be explored in more detail in future studies.

## Data Availability

The data that support the findings of this study are available from the corresponding authors on reasonable request.
